# A Novel Phenolic From the Heartwood of *Cotinus coggygria* Scop. With Potential Neuroprotective Effects: In Silico and In Vitro Studies

**DOI:** 10.1002/cbdv.71890

**Published:** 2026-11-01

**Authors:** Caiwen Meng, Yi He, Zijia Wang, Hongyun Bai, Ruyi Wang, Xiaoliang Wang, Chunlai Zhao, Meichen Li

**Affiliations:** ^1^ State Key Laboratory of Druggability Evaluation and Systematic Translational Medicine Tianjin Institute of Pharmaceutical Research Tianjin P. R. China

**Keywords:** Anacardiaceae, *Cotinus coggygria* Scop, Cotinusin A, heartwood, neuroprotective potential

## Abstract

Seven known phenolic compounds (**2**–**8**) along with one new phenolic compound (**1**) were isolated from the heartwood of *Cotinus coggygria* Scop. (Anacardiaceae). Among them, compounds **3**–**5** are reported from the genus *Cotinus* for the first time, while compounds **2** and **6**–**8** are reported from the family Anacardiaceae for the first time. The structure of the new compound was elucidated by detailed spectroscopic analysis, including HR‐ESI‐MS, 1D, and 2D NMR experiments. In vitro assays showed that compound **1** attenuated glutamate‐induced oxidative injury in HT22 cells (EC_50_ = 3.852 µM, MTS assay) and inhibited TNF‐α‐induced NF‑κB activation in 293T luciferase reporter cells. Molecular docking suggested that compound **1** may bind to IKKβ, consistent with the observed NF‑κB inhibition. Preliminary structure‐activity relationship trend analysis indicated that compounds possessing a 6″‐*O*‐rhamnose‐containing disaccharide moiety (**6**–**8**) showed stronger apparent protective activity than the 6″‐*O* ‐galloyl derivatives (**1**–**5**) under the present assay conditions. These preliminary findings suggest that cotinusin A (**1**) may warrant further investigation for its potential against glutamate‐induced oxidative neuronal injury. However, whether the observed NF‑κB inhibitory activity translates into anti‐neuroinflammatory effects in relevant models remains to be determined, and further mechanistic and in vivo studies are needed to establish its therapeutic potential.

## Graphical abstract

A new phenolic glycoside, cotinusin A, was isolated from the heartwood of *Cotinus coggygria* together with seven known compounds. Cotinusin A attenuates glutamate‑induced oxidative injury in HT22 cells (EC_50_ = 3.852 µM) and suppresses TNF‑α‑induced NF‑κB activation in a 293T luciferase reporter assay. These preliminary observations warrant further mechanistic and in vivo investigation to explore its potential against oxidative neuronal injury.

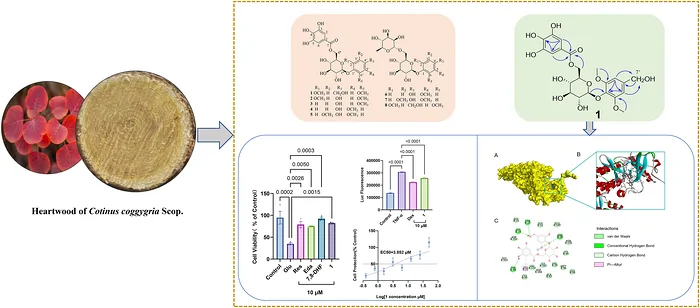

## 1 Introduction

*Cotinus coggygria* Scop. (Anacardiaceae, syn *Rhus cotinus* L.), widely known as “smoke tree” or Huanglu in traditional Chinese medicine, is a well‑recognized medicinal and ornamental plant distributed across China, Europe, and the Mediterranean region [1]. As documented in ancient herbal classics including *Ben Cao Gang Mu* and *Ben Cao Shi Yi*, various parts of *C. coggygria* (roots, stems, leaves, and heartwood) have long been used to alleviate hepatic disorders, clear heat and detoxify, promote blood circulation, and relieve inflammatory conditions [2, 3]. Modern phytochemical investigations have revealed that *C. coggygria* is rich in phenolic acids [4, 5] and flavonoids [6, 7], which are believed to contribute to its broad pharmacological activities.

Increasing evidence indicates that oxidative stress [8], neuroinflammation [9], and glutamate‐induced neuronal injury [10] are core pathological mechanisms underlying neurodegenerative diseases. In mature neurons, excessive glutamate exposure triggers overactivation of glutamate receptors, leading to calcium overload, mitochondrial dysfunction, and ultimately neuronal cell death [11]. However, undifferentiated HT22 cells lack these receptors, and glutamate‐induced injury in this model is primarily non‐receptor oxidative/oxytotic rather than classical receptor‐mediated excitotoxicity. Additionally, overactivated NF‑κB signaling‑mediated inflammatory responses exacerbate neuronal damage and accelerate disease progression [12]. Notably, extracts and phenolic constituents from *C. coggygria* exert potent free radical‑scavenging and anti‑inflammatory activities, suggesting promising potential for neuroprotection [13, 14, 15]. However, most studies have focused on crude extracts or known flavonoids, while systematic phytochemical and pharmacological investigations on phenolic compounds from this species remain limited.

In the present study, eight phenolic compounds were isolated and identified from *C. coggygria*. Among them, compound **1** was undescribed. Three known compounds (**3**–**5**) were isolated from the genus *Cotinus* for the first time, and four known compounds (**2**, **6**–**8**) were obtained from the family Anacardiaceae for the first time. The structure of the new compound was elucidated by HR‐ESI‐MS and comprehensive NMR analyses.

The protective effect of the new compound against glutamate‐induced oxidative injury was evaluated in HT22 cells, and its EC_50_ value (3.852 µM) was determined. Additionally, its inhibitory effect on TNF‐α‐induced NF‑κB activation was assessed using a luciferase reporter assay and supported by molecular docking against IKKβ. A preliminary structure‑activity‑relationship trend analysis was also conducted based on the activities of all isolated compounds. This study enriches the chemical diversity of *C. coggygria* and provides a basis for further exploration of this plant as a source of compounds with potential against oxidative neuronal injury.

## 2 Results and Discussion

### 2.1 Structural Elucidation

Compound **1** (Figure 1) was obtained as a white powder. Its HR‐ESI‐MS spectrum exhibited a molecular ion peak at *m/z* 497.1261 [M─H]^−^ (calcd for C_22_H_25_O_13_^−^: 497.1301), consistent with a molecular formula of C_22_H_26_O_13_ and 10 degrees of unsaturation. The ^1^H and ^1^ ^3^C NMR data indicated the presence of a galloyl group [*δ*_H_ 6.98 (2H, s); *δ*_C_ 110.2 (C‐2/6), 121.5, 139.7, 146.4 (C‐3/5), 168.2] and a *β*‐form glucosyl moiety [*δ*_H_ 4.82 (d, *J* = 7.6 Hz); *δ*_C_ 105.1]. In addition, the remaining ^1^H NMR signals of compound **1** revealed two methoxy groups [*δ*_H_ 3.76 (6H, s)], one oxymethylene group [*δ*_H_ 4.50 (2H, s)], and two aromatic protons [*δ*_H_ 6.60 (2H, s)]. Combined with the corresponding carbon signals from the HSQC spectrum, this fragment was found to be almost identical to the aromatic moiety of compound **8**. Furthermore, HMBC correlations were observed between H‐6″ [*δ*_H_ 4.41 (m)] of glucose and the carbonyl carbon (*δ*_C_ 168.2) of the galloyl group, as well as between H‐1″ (*δ*_H_ 4.82 (d, *J* = 7.6 Hz)) of glucose and C‐1′ (*δ*_C_ 134.8) of the aromatic ring (Figure 2). These observations confirmed that the glucose was esterified with the galloyl group at C‐6″ and linked to the aromatic moiety at C‐1″. Accordingly, the structure of compound **1** was established and named cotinusin A.

**FIGURE 1:**
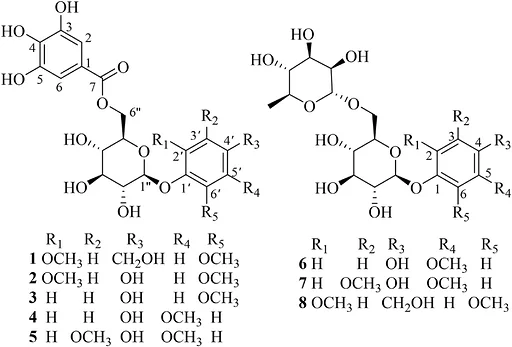
Chemical structures of isolated compounds (**1**–**8**) from the heartwood of *C. coggygria*.

**FIGURE 2:**
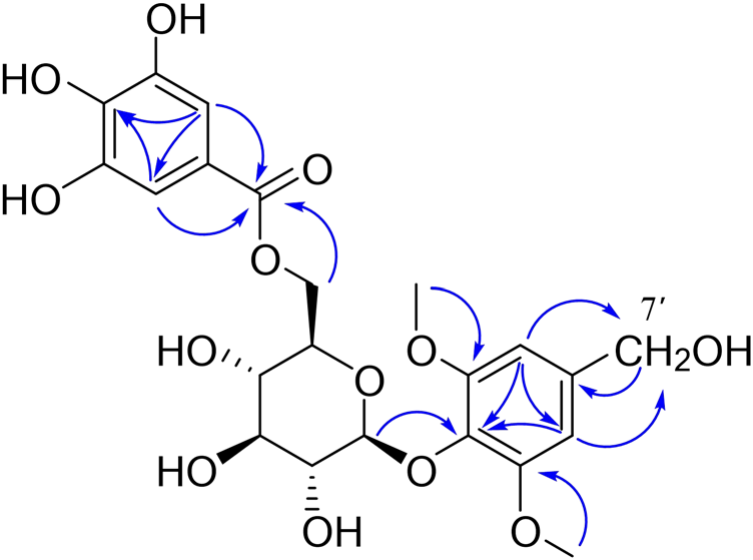
The key HMBC correlations of compound **1**.

The chemical structures of the known compounds were elucidated using NMR spectroscopy and comparisons with data from previously published literature. The known compounds were identified as 4‐hydroxy‐2,6‐dimethoxyphenol 1‐*O*‐*β*‐*D*‐(6′‐*O*‐galloyl) glucoside (**2**) [16], 3‐methoxy‐4‐hydroxyphenol‐(6′‐*O*‐galloyl)‐1‐*O*‐*β*‐*D*‐glucopyranoside (**3**) [17], methoxy‐4‐hydroxyphenol 1‐*O*‐*β*‐*D*‐(6′‐*O*‐galloyl) glucopyranoside (**4**) [18], 3,5‐dimethoxy‐4‐hydroxyphenol 1‐*O*‐*β*‐*D*‐(6′‐*O*‐galloyl) glucopyranoside (**5**) [18], cuneataside D (**6**) [19], 3,5‐dimethoxy‐4‐hydroxyphenyl 1‐*O*‐rutinoside (**7**) [19], and rhyncoside D (**8**) [20].

### 2.2 Effects of Compound **1** on Glutamate‐Induced Oxidative Injury and TNF‐α‐Induced NF‐κB Activation

It should be noted that undifferentiated HT22 cells lack ionotropic glutamate receptors; therefore, the glutamate‐induced HT22 cell injury model used here primarily reflects non‐receptor oxidative/oxytotic injury rather than classical excitotoxicity. Prior to evaluating the protective activity, the cytotoxicity of compound **1** alone was assessed in HT22 cells. As shown in Supplementary Figure S21, compound **1** exhibited low apparent cytotoxicity, with an apparent IC_50_ value of 112.6 µM. Because the same MTS‐based method was used for both cytotoxicity and protection measurements, potential interference of compound **1** with the MTS/PMS system cannot be excluded. An orthogonal cytotoxicity assay was not performed; therefore, the low apparent cytotoxicity observed by MTS should be interpreted with caution. In the glutamate‐induced injury model, compound **1** at 12.5 µM restored cell viability to 91.01% ± 1.59% of the control (Figure S22), and a concentration of 10 µM was therefore selected for subsequent mechanistic studies.

Two in vitro models were applied to evaluate the protective effect of cotinusin A (**1**) against glutamate‐induced oxidative injury and its inhibitory effect on TNF‐α‐induced NF‑κB activation. The glutamate‐induced HT22 cell injury model was used to assess protection against glutamate‐induced oxidative injury [21]. The TNF‐α‐induced NF‑κB luciferase reporter assay was employed to assess the inhibitory effect on NF‑κB transcriptional activity [22].

For the glutamate‐induced oxidative injury assay, HT22 cells were exposed to glutamate alone or co‐treated with cotinusin A (**1**) and other positive drugs [resveratrol (Res), edaravone (Eda), 7,8‐dihydroxyflavone (7,8‐DHF)] for 24 h. Glutamate treatment significantly decreased cell viability (adjusted *p* = 0.0002 vs. control). Cotinusin A (**1**) significantly attenuated glutamate‐induced oxidative injury (adjusted *p* = 0.0015 vs. model, Figure 3A). Resveratrol (adjusted *p* = 0.0026 vs. model), edaravone (adjusted *p* = 0.0050 vs. model), and 7,8‐DHF (adjusted *p* = 0.0003 vs. model) also showed protective effects.

**FIGURE 3:**
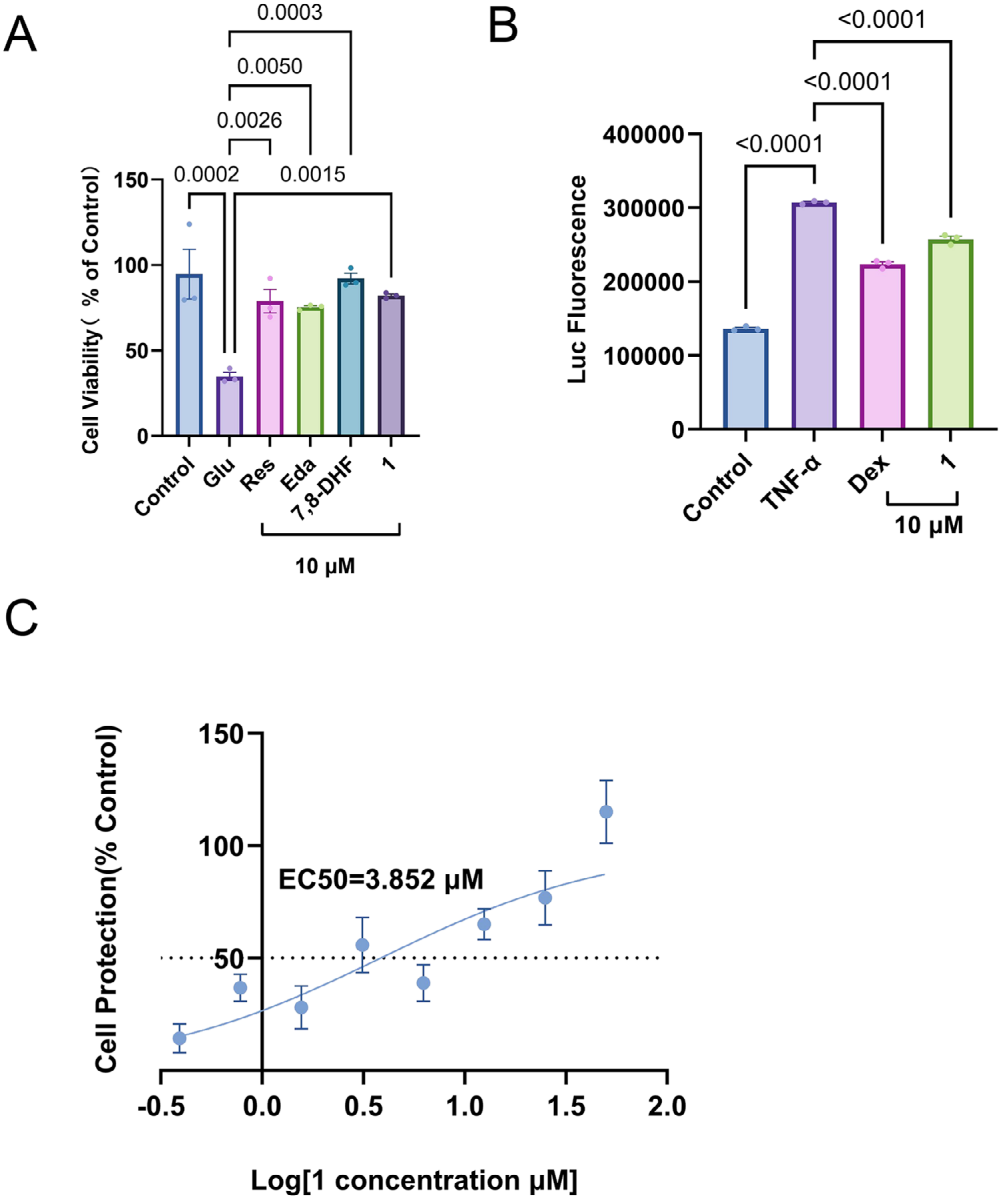
Effects of compound **1** on glutamate‐induced oxidative injury in HT22 cells and TNF‐α‐induced NF‑κB activation. (A) HT22 cells were co‐treated with Glu and Res, Eda, 7,8‐DHF, or compound **1** (10 µM) for 24 h. Cell viability was determined by MTS assay. (B) 293T cells were pretreated with Dex or compound **1** (10 µM) for 1 h, followed by stimulation with TNF‐α (10 ng/mL) for 6 h. NF‑κB promoter activity was measured using a luciferase reporter assay. (C) Concentration‑response curve of compound **1** against glutamate‐induced oxidative injury in HT22 cells, with an EC_50_ value of 3.852 µM. Data are presented as mean ± SEM (*n* = 3 independent biological experiments). Individual data points are shown. For (A) and (B), comparisons of each treatment group with the model group (Glu in A; TNF‐α in B) were performed by one‐way ANOVA followed by Dunnett's multiple comparison test. No all‐pairwise comparisons were performed. Exact adjusted *p*‐values are shown in the figure.

To determine the potency of compound **1**, a concentration‑response experiment was performed in HT22 cells against glutamate‐induced oxidative injury. As shown in Figure 3C, compound **1** displayed an overall upward trend in protective activity against glutamate‐induced oxidative injury across the tested concentration range, with an EC_50_ value of 3.852 µM determined by nonlinear regression analysis. Additionally, the protective activities of all isolated compounds (**1**–**8**) at 10 µM against glutamate‐induced oxidative injury were compared in the same model. As shown in Figure S23, all compounds, including the positive controls edaravone and 7,8‐DHF, showed a trend toward higher cell viability than the model group, although none of these differences reached statistical significance under the present assay conditions. This may be attributed to the relatively mild glutamate‐induced injury in this experiment (adjusted *p* = 0.0155 for model vs. control) compared with Figure 3A (adjusted *p* = 0.0002), which reduced the sensitivity for detecting protective effects. Despite the lack of statistical significance, the mean cell viability values suggested that compounds **6** and **7** were the most effective, followed by compound **8**, whereas compounds **4** and **5** showed the weakest effects. Based on the concentration‐response profile of compound **1** and the apparent IC_50_/EC_50_ ratio (≈ 29) obtained in the same MTS‐based assay, a concentration of 10 µM was selected for subsequent mechanistic studies. This ratio is reported as an apparent selectivity index and should not be considered a validated safety margin.

Furthermore, the inhibitory effect of cotinusin A (**1**) on TNF‐α‐induced NF‑κB activation was assessed using a TNF‐α‐induced NF‑κB luciferase reporter assay in 293T cells, which are widely employed as a standard host for reporter gene assays due to their high transfection efficiency and robust luciferase expression [22]. Cells were pretreated with dexamethasone (Dex) or cotinusin A (**1**) for 1 h, followed by stimulation with TNF‐α (10 ng/mL) for 6 h. As depicted in Figure 3B, TNF‐α stimulation remarkably increased NF‑κB activity (*p* < 0.0001 vs. control). Notably, cotinusin A (**1**) significantly suppressed NF‑κB activation (*p* < 0.0001 vs. model), and its inhibitory effect was comparable to that of dexamethasone (*p* < 0.0001 vs. model).

TNF‐α stimulation activates the NF‑κB signaling pathway primarily through the IκB kinase (IKK) complex, in which IKKβ plays a central role in phosphorylating IκBα, leading to its degradation and subsequent nuclear translocation of NF‑κB [23]. To further explore the mechanism underlying the NF‑κB inhibitory activity, molecular docking was performed against IKKβ (PDB: 4KIK), a key upstream kinase in the NF‑κB pathway. The docking protocol was validated by re‑docking the co‑crystallized ligand KSA, which reproduced the crystallographic binding pose with an RMSD of 0.955 Å (Table S1 and Figure S24). Compound **1** showed a favorable binding affinity toward IKKβ with a predicted binding affinity of −8.2 kcal/mol. As shown in Figure 4, compound **1** occupied the ATP‑binding pocket of IKKβ, forming hydrogen bonds with residues THR23, GLY24, ASN28, TYR98, GLY102, and ASP166, as well as hydrophobic interactions with surrounding residues including LEU21, VAL29, MET96, CYS99, VAL152, and ILE165. Although the predicted binding affinity of compound **1** was lower than that of the native ligand KSA (−11.9 kcal/mol), this similar binding mode suggests that compound **1** may be a putative IKK‑β‑binding candidate, consistent with the NF‑κB inhibitory activity observed in the luciferase reporter assay.

**FIGURE 4:**
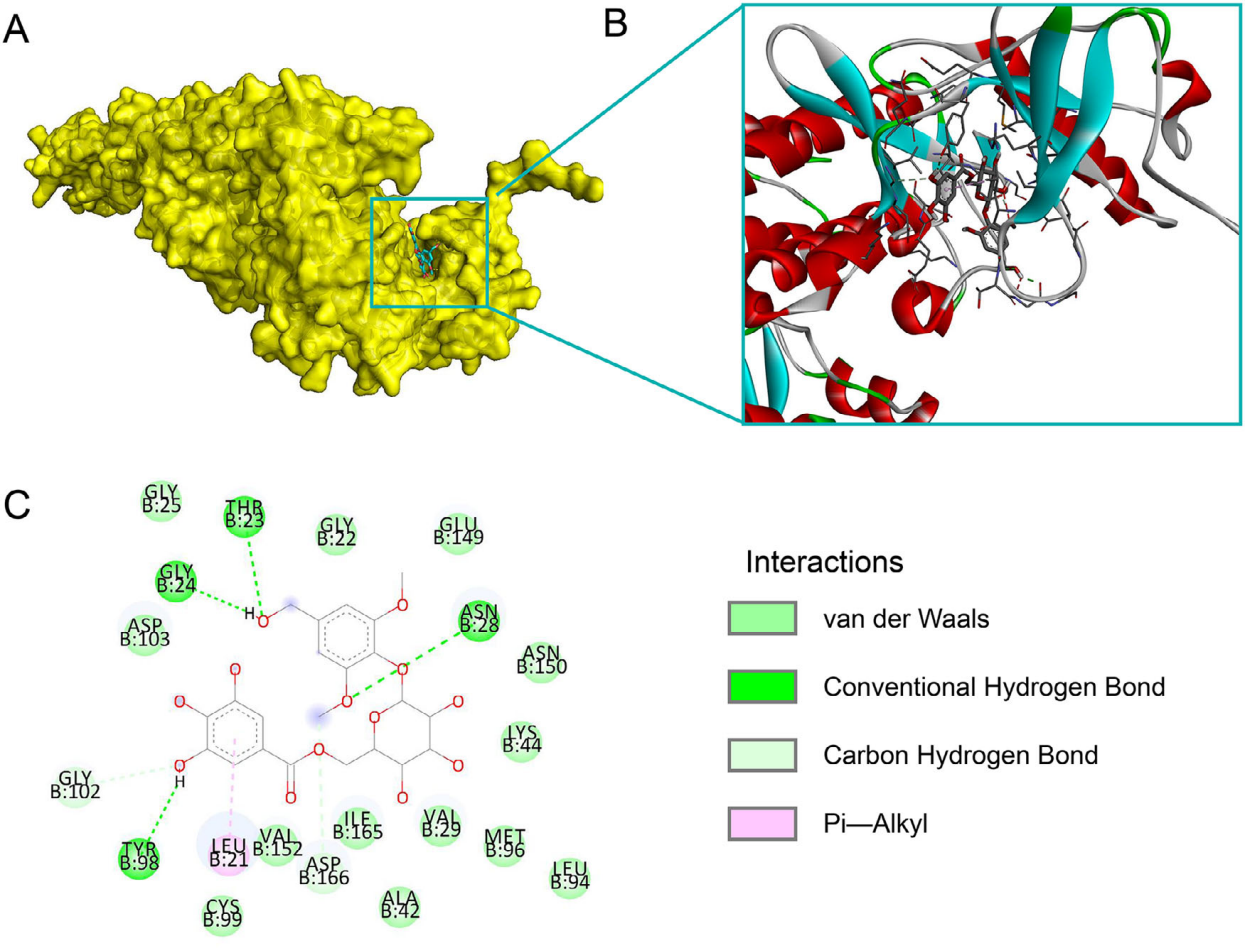
Molecular docking analysis of compound **1** with IKKβ. (A) Overall surface representation of the target protein, the ligand is located in the binding pocket. (B) Zoom‑in cartoon view showing the ligand bound within the binding pocket. (C) Two‑dimensional protein‑ligand interaction diagram.

Because both cytotoxicity and protective activity were measured using the same MTS assay, and potential interference of compound **1** with the MTS/PMS system cannot be excluded, the activities reported below are referred to as apparent activities under the conditions tested. As noted in Figure S23, none of the compounds reached statistical significance. Therefore, the following structure–activity relationship discussion is based on mean trends and should be considered preliminary. A prominent difference was observed at the 6″‑position of the central glucose moiety. Compounds **1**–**5** carrying a 6″‑*O*‑galloyl group showed weak or undetectable protection, whereas compounds **6**–**8** possessing a 6″‑*O*‑rhamnose‐containing disaccharide showed stronger protection in the MTS assay. This series‐level correlation may suggest that the glycosidic moiety contributes to the apparent activity; however, because the aglycone structures also differ among these compounds, causality cannot be established. The rhamnose‐containing disaccharide scaffold was associated with higher apparent protective activity in this series under the present assay conditions, whereas galloyl‐ester substitution was associated with weaker activity. Among compounds **6**–**8**, compounds **6** and **7** showed the strongest protection. These two analogs differ only at the R_2_ position (H vs. OCH_3_), suggesting that methoxylation at R_2_ is tolerated and does not impair protection in this assay.

Compound **8** exhibited moderate protection; however, it differs from **6** and **7** at four aromatic‐ring positions (R_1_, R_3_, R_4_, and R_5_) simultaneously. Given the multiple concurrent structural variations, the current compound set cannot establish any causal link between individual aromatic substituents and the observed activity change for compound **8**. Within the galloyl‐substituted series (**1**–**5**), compounds **1**–**3** afforded marginal protection, whereas compounds **4** and **5** showed no detectable activity. Because multiple aromatic positions differed among these analogs, we refrained from assigning activity loss to any single substituent. Collectively, these data reveal a series‐level correlation: within the tested phenolic glycosides, compounds possessing a 6″‐*O*‐rhamnose‐containing disaccharide moiety (**6**–**8**) showed stronger apparent protection than the 6″‐*O*‐galloyl derivatives (**1**–**5**) under the present assay conditions. However, because the aglycone structures also differ, this correlation does not establish that the disaccharide moiety is required for or causally responsible for the activity. The glycosidic moiety may also affect pharmacokinetic properties such as solubility, permeability, cellular uptake, and metabolic stability rather than directly interacting with the pharmacological target. Further studies using aglycone controls, sugar‐modified analogs, and target‐binding or uptake assays are needed to clarify the role of the glycosidic moiety. Conclusions on individual aromatic substituents would also require targeted single‐site analogs.

## 3 Conclusions

In summary, eight phenolic compounds were isolated and identified from the heartwood of *C. coggygria*, including one new compound, cotinusin A (**1**). Among the known isolates, compounds **3**–**5** are reported from the genus *Cotinus* for the first time, and compounds **2** and **6**–**8** are reported from the family Anacardiaceae for the first time, expanding the known chemical diversity of this species. However, because this study examined only one species and one population, and no comparative or quantitative profiling across related taxa was performed, these findings do not establish chemotaxonomic significance or identify chemotaxonomic markers. Further comparative studies across multiple *Cotinus* and Anacardiaceae species are needed to evaluate their chemophenetic value. Biological evaluation revealed that cotinusin A (**1**) attenuated glutamate‐induced oxidative injury in HT22 cells, with efficacy comparable to that of resveratrol and edaravone under the same conditions. In addition, cotinusin A (**1**) inhibited TNF‐α‐stimulated NF‑κB activation in 293T reporter cells, with potency similar to that of dexamethasone. These preliminary findings suggest that cotinusin A (**1**) may warrant further mechanistic and in vivo studies to evaluate its potential against glutamate‐induced oxidative neuronal injury. Moreover, whether the observed NF‑κB inhibitory activity translates into anti‐neuroinflammatory effects in relevant cell models remains to be determined. It should be noted that cotinusin A (**1**) occurs at low natural abundance in *C. coggygria* heartwood; only 5 mg was obtained from 20 kg of dried plant material, and direct isolation from the natural source cannot support large‑scale preparation. Further synthetic or biosynthetic efforts will be required to overcome this supply bottleneck if larger quantities are demanded for subsequent advanced pharmacological studies. This study also lays a foundation for further research into the potential of *C. coggygria* as a source of compounds with potential against oxidative neuronal injury.

## 4 Experimental Section

### 4.1 General Experimental Procedures

The NMR spectra were acquired in Methanol‐d4 using Bruker ARX‐400 spectrometers (RRID: SCR_017706), with tetramethylsilane (TMS) as an internal standard. HPLC analysis was performed on a P3100 semi‐preparative HPLC system (Elite, China), which was equipped with a Cosmosil chromatographic column (20 mm I.D. × 250 mm, 5 µm; Nacalai Tesque Inc., Japan), a UV3100 detector, and connected to an S3100 workstation. The mobile phase was delivered at a constant flow rate of 5 mL/min. The common wavelengths used were 210 and 254 nm. The purity of compound **1** was determined by analytical HPLC on a Cosmosil 5C_18_‐MS‐II column (4.6 mm I.D. × 250 mm, 5 µm; Nacalai Tesque Inc., Japan) using the same P3100 HPLC system equipped with a UV3100L detector. The mobile phase consisted of A (MeOH) and B (H_2_O) using the following gradient: 0–15 min, 20%–50% A; 15–20 min, 50%–90% A; 20–21 min, 90%–20% A; 21–25 min, 20% A. The flow rate was 1.0 mL/min, the detection wavelength was 254 nm, and the injection volume was 40 µL. The retention time of compound **1** was 11.37 min. Purity was calculated by area normalization at 254 nm and was 98.7%. A Rotavapor R‐300 (Büchi, Switzerland, RRID: SCR_019093) was used for rotary evaporation. ODS (ODS‐A‐50 µm, 120 A pore size, YMC Corporation) and Sephadex LH‐20 (Cytiva, 17‐0090‐02, RRID: AB_776969) were employed for chromatographic separation. Column chromatography was performed on silica gel (200–300 mesh, Marine Chemical Industry, Qingdao), and TLC was performed on pre‐coated silica gel GF254 plates (Marine Chemical Industry, Qingdao), with visualization under UV light (254/365 nm) or by spraying with 10% H_2_SO_4_ followed by heating. GC analyses were performed using an Agilent GC 6890 instrument (RRID: SCR_019094) on an HP‐5 column (320 µm × 30 m, 0.25 µm, Agilent, RRID: SCR_019094).

HT22 cells (RRID: CVCL_0321) were obtained from the Cell Bank of the Chinese Academy of Sciences (Catalog No. GNM47). 293T‑NF‑κB‑Luc2 reporter cells (RRID: CVCL_XY95) were purchased from Shanghai Yaji Biological (Catalog No. YW‐1533). Dulbecco's modified Eagle's medium (DMEM, Gibco, Cat. No.: C11995500BT, RRID: SCR_008458) and fetal bovine serum (FBS, Gibco) were purchased from Gibco. CellTiter 96 Aqueous One Solution Cell Proliferation Assay (MTS kit, Promega, Cat. No.: G3581, RRID: SCR_006733) was obtained from Promega (Madison, WI, USA). Dexamethasone (MCE, Cat. No.: HY‐14648, RRID: SCR_016885), resveratrol (MCE, Cat. No.: HY‐B0099, RRID: SCR_016885), and 7,8‐dihydroxyflavone (Selleck, Cat. No.: S8319, RRID: SCR_016823) were purchased from Selleck. Dimethyl sulfoxide (DMSO, Sigma–Aldrich, Cat. No.: D2650, RRID: SCR_018075) was purchased from Sigma–Aldrich (Saint Louis, MO, USA). Dulbecco's phosphate‐buffered saline (D‐PBS, Solarbio, Cat. No.: D1040, RRID: SCR_018532) was purchased from Solarbio (Beijing, China). L‐Glutamic acid (Beyotime, Cat. No.: ST1482, RRID: SCR_016986) was purchased from Beyotime Biotechnology (Shanghai, China). Recombinant human TNF‐α (MCE, Cat. No.: HY‐P78218, RRID: SCR_016885) was obtained from MedChemExpress (Monmouth Junction, NJ, USA). Penicillin‐streptomycin solution (Solarbio, Cat. No.: P1400, RRID: SCR_018532) and 0.25% trypsin‐EDTA solution (Gibco, Cat. No.: 12604021, RRID: SCR_008458) were purchased from Gibco and Solarbio, respectively.

### 4.2 Plant Material

The heartwood of *C. coggygria* was collected from the Daba Mountain area of Ankang, Shaanxi Province, China (109°01'45'' E; 32°41'05'' N) on September 28, 2024. The plant was identified by Dr. Jianyu Liu (Associate Professor, Shenyang Pharmaceutical University) and specimen (accession number: HL‐2024‐001) was deposited in the Herbarium of the State Key Laboratory of Druggability Evaluation and Systematic Translational Medicine, Tianjin Institute of Pharmaceutical Research.

### 4.3 Extraction and Isolation

The heartwood of *C. coggygria* (20 kg) was dried and pruned, then extracted with 70% EtOH (100 L each time, 3 h per extraction, repeated 3 times). The extract was evaporated under reduced pressure to yield 2023 g of crude extract. The crude extract was suspended in 5 L of warm water, followed by sequential partition extraction with 5 L of petroleum ether, 5 L of EtOAc, and 5 L of *n*‐BuOH in equal volumes to the aqueous phase, with each solvent being used for three successive extractions. This process afforded a petroleum ether fraction (14 g), an EtOAc fraction (1624 g), an *n*‐BuOH fraction (203 g), and a water fraction (182 g), respectively.

The *n*‐BuOH extract (203 g) was fractionated by silica gel column chromatography (CC) using a CH_2_Cl_2_/MeOH gradient elution (100:0 to 0:100, *v/v*), yielding ten fractions (Fr.B1–Fr.B10). Fr.B7 (19 g) was subjected to gradient elution by ODS CC using MeOH/H_2_O (5% to 100%, *v/v*), yielding 12 subfractions (Fr.B7a–Fr.B7l). Subfraction Fr.B7f (3 g) was further separated by ODS CC under the same MeOH/H_2_O gradient conditions, affording 5 subfractions (Fr.B7fa–Fr.B7fe). Fr.B7fb (0.2 g) was purified by semipreparative HPLC with an isocratic elution of MeOH/H_2_O (20:80, *v/v*), yielding compound **1** (*t*_R_ = 101 min; 5 mg). Fr.B7fd (0.3 g) was purified by semipreparative HPLC with an isocratic elution of MeOH/H_2_O (20:80, *v/v*), yielding compound **4** (*t*_R_ = 53 min; 3 mg) and compound **5** (*t*_R_ = 61 min; 40 mg); elution with an isocratic elution of ACN/H_2_O (10:90, *v/v*) afforded compound **3** (*t*_R_ = 59 min; 3.5 mg).

Fr.B8 (30 g) was fractionated by gradient elution by ODS CC using MeOH/H_2_O (5% to 100%, *v/v*), resulting in 14 subfractions (Fr.B8a–Fr.B8n). Subfraction Fr.B8c (1.2 g) was further separated by ODS CC under the same MeOH/H_2_O gradient conditions, affording 8 subfractions (Fr.B8ca–Fr.B8ch). Fr.B8cd (0.2 g) was purified by semipreparative HPLC with an isocratic elution of MeOH/H_2_O (12:88, *v/v*), yielding compound **6** (*t*_R_ = 37 min; 20 mg) and compound **7** (*t*_R_ = 46 min; 21 mg). Fr.B8d (2.1 g) was further separated by ODS CC under the same MeOH/H_2_O gradient conditions to give 9 subfractions (Fr.B8da–Fr.B8di). Compound **8** (*t*_R_ = 35 min; 15 mg) was obtained from subfraction Fr.B8db (0.2 g) by semipreparative HPLC using an isocratic elution of MeOH/H_2_O (17:83, *v/v*). Subfraction Fr.B8f (1.8 g) was further separated by ODS CC under the same MeOH/H_2_O gradient conditions, affording 7 subfractions (Fr.B8fa–Fr.B8fg). Fr.B8fd (0.3 g) was purified by semipreparative HPLC with an isocratic elution of MeOH/H_2_O (25:75, *v/v*), yielding compound **2** (*t*_R_ = 31 min; 5 mg).

Cotinusin A (**1**): white amorphous powder; ^1^H and ^13^C NMR spectroscopic data, see Table 1; HRESIMS (negative‐ion mode) m/z 497.1261 [M─H]^−^ (calcd. for C_22_H_25_O_13_, 497.1301). The purity of compound **1** was determined to be 98.7% by HPLC analysis (Figure S1). NMR and MS spectra are displayed in Figures S2–S6.

**TABLE 1:** ^1^H NMR, ^13^C NMR, and HMBC spectroscopic data for compound **1**.

Nos_._	*δ*_H_	*δ*_C_	HMBC (H to C)
1	—	121.5	—
2/6	6.98 (s)	110.2	110.2, 121.5, 139.7, 146.4, and 168.2
3/5	—	146.4	—
4	—	139.7	—
7	—	168.2	—
1′	—	134.8	—
2′/6′	—	154.4	—
3′/5′	6.60 (s)	105.5	65.2, 105.5, 134.8, and 154.4
4′	—	139.8	—
7′	4.50 (s)	65.2	105.5, 139.8
─OCH_3_	3.76 (s)	56.8	154.4
1″	4.82 (d, *J* = 7.6 Hz)	105.1	77.8, 134.8
2″	3.47 (m)	75.7	71.9
3″	3.48 (m)	77.8	75.6
4″	3.46 (m)	71.9	77.8
5″	3.45 (m)	75.6	75.7
6″	4.41 (m)	64.6	168.2

The NMR data for known compounds (**2**–**8**) are provided in the (Figures S7–S20).

### 4.4 Acid Hydrolysis

Acid hydrolysis of compound **1** was performed as previously described [24]. Briefly, the hydrolyzed monosaccharides were derivatized with L‐cysteine methyl ester hydrochloride and trimethylchlorosilane, then analyzed by GC on an HP‐5 column. D‐glucose was confirmed by comparison with the authentic standard.

### 4.5 Cell Viability Assay

HT22 cells were seeded at a density of 1 × 10^5^ cells per well in 96‐well plates and incubated overnight at 37°C in a humidified atmosphere with 5% CO_2_. The cells were then co‐treated with 30 mM glutamate and test compounds (10 µM) for 24 h. After incubation, cell viability was determined by MTS assay. Briefly, MTS reagent was added to each well, and the plates were incubated for an additional 2 h at 37°C. The absorbance at 490 nm was measured using a microplate reader.

### 4.6 Luciferase Reporter Assay

293T cells were seeded at 3 × 10^4^ cells per well in 96‐well plates and incubated overnight. The cells were pretreated with test compounds (10 µM) for 1 h, followed by stimulation with 10 ng/mL TNF‐α for 6 h. Luciferase activity was measured using a luciferase reporter assay system according to the manufacturer's instructions, and the luminescence intensity was detected with a multimode microplate reader.

### 4.7 Statistical Analysis

Statistical analysis was performed using GraphPad Prism 10. Data are presented as mean ± SEM. *n* = 3 independent biological experiments. For comparisons of each treatment group with the injury control, one‐way ANOVA followed by Dunnett's multiple comparison test was used. No all‐pairwise comparisons were performed. Technical replicates were averaged before analysis. Normality and homoscedasticity were assessed, and the assumptions for one‐way ANOVA were considered appropriate. Exact adjusted *p*‐values are shown in the figures. Differences were considered statistically significant at *p* < 0.05.

### 4.8 In Silico Molecular Docking

Molecular docking was performed to predict the binding mode of compound **1** with IKKβ (PDB ID: 4KIK). The crystal structure was retrieved from the RCSB Protein Data Bank (https://www.rcsb.org/).

Protein preparation was carried out using AutoDockTools version 1.5.7 (distributed as part of MGLTools). All water molecules and heteroatoms were removed from the protein structure, polar hydrogens were added, and Kollman charges were assigned. The three‐dimensional structure of compound **1** was built using ChemDraw and energy‐minimized using the MMFF94 force field. The ligand was then prepared in AutoDockTools by adding Gasteiger charges, defining rotatable bonds, and saving in PDBQT format [25].

Molecular docking simulations were performed using AutoDock Vina [26]. The grid box was centered at the binding pocket of the co‐crystallized ligand KSA (coordinates: −14.04, −31.93, and −73.44 Å), with a grid spacing of 0.375 Å and dimensions of 40 × 40 × 40 Å. The exhaustiveness value was set to 8. The co‐crystallized ligand KSA was used as a reference compound.

To validate the docking protocol, KSA was re‐docked into the binding site of IKKβ using the same parameters. The re‐docked pose reproduced the crystallographic conformation with a root‐mean‐square deviation (RMSD) of 0.955 Å (Table S1 and Figure S24), confirming the reliability of the docking procedure. Binding poses were ranked by predicted binding affinity (kcal/mol). Protein–ligand interactions, including hydrogen bonds and hydrophobic interactions, were visualized and analyzed using Discovery Studio Visualizer.

## Author Contributions

Caiwen Meng and Hongyun Bai were responsible for the chemical isolation and structural elucidation, with Caiwen Meng additionally taking charge of the initial drafting of the manuscript. Yi He, Zijia Wang, and Ruyi Wang conducted the bioassays for biological activity testing. Meichen Li, Chunlai Zhao, and Xiaoliang Wang were responsible for the funding, supervised the project, and contributed to the revision of the manuscript.

## Conflicts of Interest

The authors declare no conflicts of interest.

## Supporting information




**Supporting File**: cbdv71890‐sup‐0001‐SuppMat.docx.

## Data Availability

The data that support the findings of this study are available in the Supporting Information of this article.
