# Supplementary material for: A Novel Phenolic From the Heartwood of Cotinus coggygria Scop. With Potential Neuroprotective Effects: In Silico and In Vitro Studies
Source: Chem Biodivers. 2026 Nov 1;23(11):e71890. doi: 10.1002/cbdv.71890 (PMC13724183; doi:10.1002/cbdv.71890)
Supplement: Supplementary file 1 [file CBDV-23-e71890-s001.docx]

**A Novel Phenolic from the Heartwood of *Cotinus coggygria* Scop. with Potential Neuroprotective Effects: In Silico and In Vitro Studies**

Caiwen Meng^#^, Yi He^#^, Zijia Wang, Hongyun Bai, Ruyi Wang, Xiaoliang Wang^*^, Chunlai Zhao^*^, Meichen Li^*^

*State Key Laboratory of Druggability Evaluation and Systematic Translational Medicine,* *Tianjin Institute of Pharmaceutical Research, Tianjin 300301, PR China*

^*^Corresponding authors:

E-mail address: wangxiaoliang@tipr.com.cn (X.L. Wang)

zhaochunlai@tipr.com.cn (C.L. Zhao)

limeichen@tipr.com.cn (M.C. Li)

**^#^** These authors contributed equally to this work and shared first authorship.

**Figures and NMR data of compounds 1-8**

Figure S1 Purity of compound **1** was detected by HPLC on a Cosmosil 5C_18_‑MS‑II column (4.6 mm i.d. × 250 mm), with UV detection at 254 nm. A: MeOH, B: H_2_O. Flow rate: 1.0 mL/min.

Figure S2 HRESIMS spectrum of compound **1**

Figure S3 ^1^H NMR spectrum (400 MHz, Methanol-*d*_4_) of compound **1**

Figure S4 ^13^C NMR spectrum (101 MHz, Methanol-*d*_4_) of compound **1**

Figure S5 HSQC spectrum of compound **1**

Figure S6 HMBC spectrum of compound **1**

**Compound 2** 4-hydroxy-2,6-dimethoxyphenol 1-*O*-*β*-*D*-(6'-*O*-galloyl) glucoside

^1^H NMR (400 MHz, Methanol-*d*_4_): *δ*_H_ 7.05 (2H, s, H-2, 6), 6.07 (2H, s, H-3′, 5′), 4.63 (1H. d, *J* = 7.4 Hz, H-1″), 4.45 (2H, m, H-6″), 3.69 (6H, s, 2′, 6′-OCH_3_), 3.49 (1H, m, H-5″), 3.31 (3H, m, H-2″, 3″, 4″); ^13^C NMR (101 MHz, Methanol-*d*_4_): *δ*_C_ 168.3 (C-7), 156.0 (C-4′), 154.9 (C-2′, 6′), 146.5 (C-3, 5), 139.8 (C-4), 129.4 (C-1′), 121.5 (C-1), 110.2 (C-2, 6), 106.3 (C-1″), 94.4 (C-3′, 5′), 77.7 (C-3″), 75.7 (C-5″), 75.6 (C-2″), 71.6 (C-4″), 64.6 (C-6″), 56.7 (2′, 6′-OCH_3_).

Figure S7 ^1^H NMR spectrum (400 MHz, Methanol-*d*_4_) of compound **2**

Figure S8 ^13^C NMR spectrum (101 MHz, Methanol-*d*_4_) of compound **2**

**Compound 3** 3-methoxy-4-hydroxyphenol-(6'-*O*-galloyl)-1-*O*-*β*-*D*-glucopyranoside

^1^H NMR (400 MHz, Methanol-*d*_4_): *δ*_H_ 7.10 (2H, s, H-2, 6), 6.94 (1H, d, *J* = 8.7 Hz, H-5′), 6.43 (1H, d, *J* = 2.7 Hz, H-2′), 6.18 (1H, dd, *J* = 8.7, 2.8 Hz, H-6′), 4.67 (1H, d, *J* = 7.3 Hz, H-1″), 4.54 (1H, dd, *J* = 11.8, 2.1 Hz, H-6″a), 4.43 (1H, dd, *J* = 11.9, 6.3 Hz, H-6″b), 3.78 (3H, s, -OCH_3_), 3.64 (1H, m, H-5″), 3.47 (3H, m, H-2″, 3″, 4″); ^13^C NMR (101 MHz, Methanol-*d*_4_): *δ*_C_ 168.3 (C-7), 154.9 (C-4′), 152.0 (C-3′), 146.5 (C-3, 5), 141.0 (C-1′), 139.9 (C-4), 121.4 (C-1), 120.5 (C-5′), 110.3 (C-2, 6), 107.7 (C-6′), 104.4 (C-1″), 101.8 (C-2′), 77.7 (C-3″), 75.6 (C-5″), 75.0 (C-2″), 71.7 (C-4″), 64.8 (C-6″), 56.5 (-OCH_3_).

Figure S9 ^1^H NMR spectrum (400 MHz, Methanol-*d*_4_) of compound **3**

Figure S10 ^13^C NMR spectrum (101 MHz, Methanol-*d*_4_) of compound **3**

**Compound 4** methoxy-4-hydroxyphenol 1-*O*-*β*-*D*-(6′-*O*-galloyl) glucopyranoside

^1^H NMR (400 MHz, Methanol-*d*_4_): *δ*_H_ 7.10 (2H, s, H-2, 6), 6.69 (1H, d, *J* = 2.7 Hz, H-2′), 6.62 (1H, d, *J* = 8.6 Hz, H-5′), 6.56 (1H, dd, *J* = 8.7, 2.6 Hz, H-6′), 4.73 (1H, d, *J* = 7.3 Hz, H-1″), 4.60 (1H, d, *J* = 12.0 Hz, H-6″a), 4.43 (1H, dd, *J* = 11.9, 6.6 Hz, H-6″b), 3.70 (3H, s, -OCH_3_), 3.46 (3H, m, H-2″, 3″, 4″); ^13^C-NMR (151 MHz, Methanol-*d*_4_): *δ*_C_ 168.3 (C-7), 152.7 (C-1′), 149.2 (C-3′), 146.6 (C-3, 5), 143.1 (C-4′), 140.1 (C-4), 121.3 (C-1), 116.1 (C-5′), 110.2 (C-6′, 2, 6), 104.0 (C-1″), 103.9 (C-2′), 77.8 (C-3″), 75.7 (C-4″), 75.0 (C-5″), 71.8 (C-2″), 65.0 (C-6″), 56.3 (-OCH_3_).

Figure S11 ^1^H NMR spectrum (400 MHz, Methanol-*d*_4_) of compound **4**

Figure S12 ^13^C NMR spectrum (101 MHz, Methanol-*d*_4_) of compound **4**

**Compound 5** 3,5-dimethoxy-4-hydroxyphenol 1-*O*-*β*-*D*-(6'-*O*-galloyl) glucopyranoside

^1^H NMR (400 MHz, Methanol-*d*_4_): *δ*_H_ 7.07 (2H, s, H-2, 6), 6.41 (2H, s, H-2′, 6′), 4.76 (1H, d, *J* = 7.6 Hz, H-1″), 4.64 (1H, d, *J* = 12.6 Hz, H-6″a), 4.43 (1H, dd, *J* = 12.0, 6.4 Hz, H-6″b), 3.74 (1H, m, H-5″), 3.66 (6H, s, 3′, 5′-OCH_3_), 3.45 (3H, m, H-2″, 3″, 4″); ^13^C-NMR (151 MHz, Methanol-*d*_4_): *δ*_C_ 168.2 (C-7), 152.2 (C-1′), 149.2 (C-3′, 5′), 146.5 (C-4), 139.8 (C-3, 5), 132.2 (C-4′), 121.3 (C-2, 6), 110.1 (C-1), 103.9 (C-1″), 96.8 (C-2′, 6′), 77.6 (C-3″), 75.7 (C-5″), 74.9 (C-2″), 71.7 (C-4″), 65.1 (C-6″), 56.6 (3′, 5′-OCH_3_).

Figure S13 ^1^H NMR spectrum (400 MHz, Methanol-*d*_4_) of compound **5**

Figure S14 ^13^C NMR spectrum (101 MHz, Methanol-*d*_4_) of compound **5**

**Compound 6** cuneataside D

^1^H NMR (400 MHz, Methanol-*d*_4_): *δ*_H_ 6.74 (1H, d, *J* = 2.7 Hz, H-5), 6.71 (1H, d, *J* = 8.7 Hz, H-2), 6.58 (1H, dd, *J* = 8.7, 2.7 Hz, H-6), 4.72 (2H, dd, *J* = 4.5, 2.8 Hz, H-1′, 1″), 4.02 (1H, dd, *J* = 10.8, 1.7 Hz, H-6′), 3.83 (3H, s, 3-OCH_3_), 1.22 (3H, d, *J* = 6.2 Hz, H-6″); ^13^C NMR (101 MHz, Methanol-*d*_4_): *δ*_C_ 152.7 (C-1), 149.2 (C-3), 143.0 (C-4), 116.1 (C-5), 110.0 (C-6), 103.9 (C-2), 103.7 (C-1′), 102.1 (C-1″), 78.0 (C-3′), 76.8 (C-5′), 74.9 (C-2′), 74.0 (C-4″), 72.4 (C-5″), 72.1 (C-2″), 71.6 (C-4′), 69.8 (C-3″), 67.9 (C-6′), 56.5 (3-OCH_3_), 18.0 (C-6″).

Figure S15 ^1^H NMR spectrum (400 MHz, Methanol-*d*_4_) of compound **6**

Figure S16 ^13^C NMR spectrum (101 MHz, Methanol-*d*_4_) of compound **6**

**Compound 7** 3,5-dimethoxy-4-hydroxyphenyl 1-*O*-rutinoside

^1^H NMR (400 MHz, Methanol-*d*_4_): *δ*_H_ 6.46 (2H, s, H-2, 6), 4.74 (1H, d, *J* = 7.3 Hz, H-1′), 4.71(1H, d, *J* = 1.7 Hz, H-1″), 4.06 (1H, m, H-6′), 3.82 (6H, s, 3, 5-OCH_3_), 1.22 (3H, d, *J* = 6.2 Hz, H-6″); ^13^C NMR (101 MHz, Methanol-*d*_4_): *δ*_C_ 152.3 (C-4), 149.4 (C-3, 5), 132.2 (C-1), 103.7 (C-1′), 102.0 (C-1″), 96.9 (C-2, 6), 77.8 (C-3′), 76.8 (C-5′), 74.9 (C-2′), 74.1(C-4″), 72.4 (C-3″), 72.1 (C-2″), 71.5 (C-4′), 69.8 (C-5″), 67.8 (C-6′), 56.9 (3, 5-OCH_3_), 18.0 (C-6″).

Figure S17 ^1^H NMR spectrum (400 MHz, Methanol-*d*_4_) of compound **7**

Figure S18 ^13^C NMR spectrum (101 MHz, Methanol-*d*_4_) of compound **7**

**Compound 8** rhyncoside D

^1^H NMR (400 MHz, Methanol-*d*_4_): *δ*_H_ 6.69 (2H, s, H-3, 5), 4.79 (1H, d, *J* = 7.6 Hz, H-1′), 4.61 (1H, d, *J* = 1.6 Hz, H-1″), 4.55 (2H, s, H-7), 3.85 (6H, s, 2, 6-OCH_3_), 1.18 (3H, d, *J* = 6.2 Hz, H-6″); ^13^C NMR (101 MHz, Methanol-*d*_4_): *δ*_C_ 154.4 (C-2, 6), 139.6 (C-4), 134.9 (C-1), 105.6 (C-3, 5), 105.1 (C-1′), 102.3 (C-1″), 77.9 (C-3′), 77.4 (C-5′), 75.9 (C-2′), 74.0 (C-4″), 72.3 (C-3″), 72.0 (C-2″), 71.9 (C-4′), 69.7 (C-5″), 68.2 (C-6′), 65.3 (C-7), 56.9 (2, 6-OCH_3_), 18.0 (C-6″).

Figure S19 ^1^H NMR spectrum (400 MHz, Methanol-*d*_4_) of compound **8**

Figure S20 ^13^C NMR spectrum (101 MHz, Methanol-*d*_4_) of compound **8**

Figure S21. Determination of apparent IC_50_ of compound **1** in HT22 cells. Cells were exposed to gradient concentrations of compound **1** for 24 h. Cell viability is shown as mean ± SEM, n = 3. Apparent IC_50_ was derived from non‑linear regression analysis.

Figure S22. Protective effect of compound **1** against glutamate-induced oxidative injury in HT22 cells. Cells were co-treated with 30 mM glutamate and compound **1** at the indicated concentrations (0.390625–50 μM; two-fold serial dilutions) for 24 h. Cell viability was determined by MTS assay. Control, untreated cells; Model, glutamate-injured cells. Data are presented as mean ± SEM (n = 3 independent biological experiments). Statistical analysis was performed using one-way ANOVA followed by Dunnett’s multiple comparison test. The model group was compared with the control to confirm glutamate-induced injury, and each compound **1**-treated group was compared with the model group. Adjusted *p* values are shown above the brackets.

Figure S23 Comparative apparent protective activities of compounds **1**–**8** (10 μM) against glutamate‑induced oxidative injury in HT22 cells. HT22 cells were co‑treated with 30 mM glutamate together with individual compounds (**1**–**8**, 10 μM), edaravone or 7,8‑DHF for 24 h. Cell viability was measured by MTS assay. Data are presented as mean ± SEM (n = 3 independent biological experiments). Individual data points are overlaid. Statistical analysis was performed using one‑way ANOVA followed by Dunnett’s multiple comparison test for comparison of each treatment group versus the glutamate‑injured model group. Due to the mild extent of glutamate‑induced cell death in this experimental batch (model vs control, adjusted *p* = 0.0155), the assay window was narrow and statistical sensitivity was low. No treatment reached statistically significant protection relative to the model group. This figure only illustrates mean cell‑viability trends.

Figure S24 Superposition of the top‑ranked docked pose (model 1, green sticks) and co‑crystallized ligand (red sticks). The RMSD value of 0.955 Å was calculated without atom superimposition in PyMOL (cycles=0, transform=0), demonstrating the validity of the docking protocol.

Table S1 Redocking validation results of the co-crystallized ligand KSA into the binding site of IKKβ (PDB: 4KIK).

| Model | Binding Affinity (kcal/mol) | RMSD (Å) |
| --- | --- | --- |
| 1 | -11.9 | 0.955 |
| 2 | -11.1 | 1.959 |
| 3 | -10.6 | 1.964 |
| 4 | -10.6 | 1.954 |
| 5 | -10.6 | 1.200 |
| 6 | -10.2 | 1.907 |
| 7 | -10.1 | 0.953 |
| 8 | -9.8 | 1.770 |
| 9 | -9.2 | 1.776 |
